# Cinobufotalin Ameliorates the Development of Pulmonary Fibrosis by Suppressing the TGF‐β/Smad Pathway via Regulating PI15

**DOI:** 10.1111/jcmm.70788

**Published:** 2025-08-22

**Authors:** Dong Xia, Xingyan Liu, Qiuting Yang, Jie Li, Li Li, Yong You, Jing Wang, Weiyi Fang, Huiling Yang

**Affiliations:** ^1^ The Affiliated Dongguan Songshan Lake Central Hospital Guangdong Medical University Dongguan China; ^2^ Department of Respiratory Medicine The Second Affiliated Hospital of Hainan Medical University Haikou China; ^3^ Department of Pulmonary and Critical Care Medicine The Second Affiliated Hospital of Guangzhou Medical University Guangzhou China

**Keywords:** cinobufotalin, EMT, PI15, pulmonary fibrosis, TGF‐β/Smad pathway

## Abstract

Pulmonary fibrosis (PF) is a common hallmark of several types of interstitial lung diseases (ILDs), for which effective therapeutic drugs are lacking. The small‐molecule chemical compound cinobufotalin (CB) has demonstrated significant anti‐cancer effects in lung cancer. In this study, we first found that CB attenuated bleomycin (BLM)‐induced PF and inhibited transforming growth factor‐beta 1 (TGF‐β1)‐induced myofibroblast activation and epithelial‐mesenchymal transition (EMT). Subsequently, comparative RNA sequencing (RNA‐Seq) was conducted to analyse the lung gene expression profiles in mice. Interestingly, peptidase inhibitor 15 (PI15) was identified as a significantly differentially expressed gene (DEG) and may be a potential target in PF progression. Mechanistic studies showed that CB exerts anti‐PF effects by inhibiting PI15 and thereby regulating the TGF‐β/Smad signalling pathway. Our data demonstrated that CB represents a promising anti‐PF drug and may be a candidate therapeutic for PF patients.

## 1 Introduction

Pulmonary fibrosis (PF) is a fatal lung disease of unknown aetiology, characterised as chronic, progressive and irreversible, whose pathological hallmark is massive abnormal fibroblast proliferation, especially myofibroblasts, and accompanied by excessive extracellular matrix (ECM) deposition [1]. Furthermore, the disease manifests cardinal symptoms including progressive dyspnea and declining lung function, ultimately resulting in poor prognosis [2]. Accumulating evidence indicates that PF pathogenesis involves interconnected pathological processes, including endoplasmic reticulum stress, proteostasis imbalance, oxidative stress, chronic inflammation, aberrant EMT activation, and myofibroblast activation [3, 4, 5]. Notably, EMT is a key process in PF, where aberrant EMT activation drives fibroblast‐to‐myofibroblast differentiation, promoting excessive ECM deposition and organ lesions [6, 7]. Consequently, inhibition of myofibroblast activation and aberrant EMT activation is an effective means to mitigate PF progression [8]. Epidemiological studies have confirmed that PF is prevalent in elderly males, with steadily rising incidence, surpassing mortality rates of numerous cancers, and that almost all patients with PF ultimately die from progressive respiratory failure [9]. Nintedanib and pirfenidone are the only FDA‐approved drugs for PF, but neither reverses established fibrosis, and they can only be used as a palliative treatment strategy to slow disease progression [10, 11]. Consequently, developing safer and more effective PF therapies represents an urgent unmet need.

CB belongs to the group of toad steroidal alkenes and was isolated from the skin secretions of the giant toad, *Bufo vulgaris* [12, 13]. Recent studies demonstrate that CB exerts significant anti‐tumour effects against diverse malignancies, including hepatocellular carcinoma, gastric cancer, and lung cancer [14, 15, 16]. A significant proportion of patients develop PF‐related complications during lung cancer therapy, and abundant epidemiological data confirm an association between PF and lung cancer [17, 18, 19]. Nevertheless, the anti‐PF mechanism of CB remains elusive.

Peptidase inhibitor 15 (PI15), a trypsin‐binding protein of the cysteine‐rich secretory proteins (CAP) superfamily, localises to the ECM and regulates ECM remodelling and tissue branching morphogenesis [20, 21]. According to reports, PI15 inhibits WNT signalling to regulate mesenchymal and chondrocyte precursor cell cohesion [22]. Notably, PI15 is upregulated in multiple tumour types, with significant overexpression in cholangiocarcinoma tissues compared to normal liver controls [23]. RNA‐Seq analysis revealed significant upregulation of PI15 expression in bleomycin (BLM)‐induced PF mice. CB treatment effectively reversed this upregulation. However, the mechanistic role of PI15 in PF progression remains to be elucidated.

This study demonstrates CB as a promising anti‐PF candidate. Using both in vivo BLM‐induced PF murine models and an in vitro TGF‐β1‐stimulated cell model, we systematically assessed the therapeutic efficacy of CB. Importantly, mechanistic investigations revealed that CB effectively inhibits the TGF‐β/Smad signalling pathway.

## 2 Materials and Methods

### 2.1 Animals and Treatments

Male Institute of Cancer Research (ICR) mice with the age of 4–5 weeks old were used in this study (Spectrum Beijing Biotechnology Co. Ltd., Beijing, China). The mice were maintained in a Specific Pathogen‐Free (SPF) environment with a 12‐h light–dark cycle and were acclimated to the conditions with at least 1 week of adaptive feeding before the experiments.

In the preventive experiment, mice were divided into 3 groups: (1) Control (saline), (2) BLM (tail vein injection), (3) BLM + CB (4 mg/kg, i.p.). The mice were administered 10 mg/kg bleomycin hydrochloride (BLM) daily via tail vein injection for 14 days (MCE, USA, HY‐17565A) to induce PF [24]. The 4 mg/kg CB was administered intraperitoneally every 3 days until the 14th day (MCE, USA, HY‐N0880), and all mice were sacrificed on the 28th day.

For the therapeutic experiment, the mice were divided into 3 groups: (1) Control (saline), (2) BLM (tail vein injection), (3) BLM + CB (4 mg/kg, i.p.). Dosing was administered intraperitoneally every 3 days starting on day 14 until day 28. On the 28th day, all mice were sacrificed and tissues were collected for further investigation.

### 2.2 Body Weight and Lung Coefficient

Mouse body weight growth curve was generated and body weights were measured and recorded. On day 28, the mice were euthanised to obtain lung wet weights and calculate lung coefficient. Lung coefficients were calculated using the formula: (lung wet weight/body weight) × 100%.

### 2.3 H&E and Masson's Trichrome Staining

Mouse lung tissues were excised, fixed, and stored in 4% paraformaldehyde for 24 h, embedded in paraffin, and sectioned at 4 μm thickness for histological examination. The sections were stained with H&E staining kit (Solarbio, China, G1120) and Masson's trichrome staining kit (Solarbio, China, G1340). All sections were examined microscopically for comparison. The Ashcroft score was applied to H&E staining to assess the degree of fibrosis [25].

### 2.4 Western Blot Analysis

Proteins were extracted from mouse lung tissues and cells using RIPA lysis buffer, followed by boiling at 98°C for 8 min to denature proteins. The BCA assay kit (TIANGEN, China, PA115) was employed to determine protein concentrations. Proteins were separated by 10% SDS‐PAGE and transferred to polyvinylidene difluoride membranes. Membranes were blocked with 5% skim milk for 1 h at room temperature, and then incubated with primary antibodies overnight at 4°C. Protein bands were visualised using a chemiluminescence imaging system.

### 2.5 Quantitative Real‐Time PCR

Total RNA was extracted from cells and lung tissues using an RNA extraction kit (Foregene, China, RE‐03014). Total RNA was reverse transcribed using a reverse transcription reagent (AG, China, AG11728) to generate cDNA. The Bio‐Rad CFX96 Real‐Time System was used for quantitative real‐time PCR (qPCR).

### 2.6 ELISA Assay

Mouse serum interleukin‐6 (IL‐6) levels were quantified using a commercial ELISA kit (LIANKE, China, EK206HS‐96) according to the manufacturer's instructions.

### 2.7 Hydroxyproline (HYP) Assay

HYP content in lung tissues was quantified using a HYP assay kit (Jiancheng, China, A030‐2‐1) according to the manufacturer's protocol. Absorbance was measured at 550 nm, and the HYP concentrations were calculated as μg HYP/mg wet tissue weight [26].

### 2.8 High‐Throughput Sequencing and Bioinformatics Analysis

Personalbio Bioscience Co. Ltd. (Shanghai, China) performed high‐throughput mRNA sequencing among three groups of mouse lung tissues, followed by bioinformatics analysis. DEGs were identified using thresholds of |log_2_FC| ≥ 1 and *p* < 0.05. Kyoto Encyclopedia of Genes and Genomes (KEGG) pathway enrichment analysis was conducted.

### 2.9 Cell Culture and Processing

MRC‐5 (Procell, China, CL‐0161) and HFL‐1 (Procell, China, CL‐0106) human lung fibroblasts were grown in MEM and Ham's F‐12 K media. The A549 (Procell, China, CL‐0016) cell line, derived from human alveolar basal epithelial cells, was grown in DMEM. Cells were harvested after 24 or 48 h of treatment with TGF‐β1 (10 ng/mL) and CB (400 nm) [27]. All cellular samples were incubated at 37°C with 5% CO_2_.

### 2.10 Cell Viability Determination

The CCK‐8 assay was used to evaluate CB cytotoxicity. Briefly, cells were plated at a density of 4000–8000 cells per well into 96‐well plates. Cells were treated with CB (0, 100, 200, 400, 600, 800, and 1000 nM) for 24 and 48 h, with or without TGF‐β1. After incubation, 10 μL CCK‐8 solution was added to assess cell viability at various drug concentrations.

### 2.11 Wound Migration Assay

To evaluate the migration ability, MRC‐5, HFL‐1, and A549 cells treated with or without TGF‐β1 and/or CB were seeded into 6‐well plates. The cell layer was scratched with a 10 μL pipette tip. The wound closure rate was measured for each group using ImageJ software after photographing the wounded region at 0, 24 and 48 h.

### 2.12 Small Interfering RNA Transfections

PI15 siRNA and negative control siRNA were transfected using Lipofectamine 3000 (Invitrogen, USA, L3000015) according to the manufacturer's instructions. Negative control siRNA and PI15 siRNA were purchased from Guangzhou RiboBio Co. Ltd. Cells were then stimulated with or without TGF‐β1, along with CB for 48 h before being harvested for further study.

### 2.13 Co‐Immunoprecipitation (Co‐IP) Assay

Total protein was extracted from MRC‐5 and HFL‐1 cells using IP lysis buffer. Cell lysates were incubated with anti‐PI15 antibody or normal IgG at 4°C overnight with gentle rotation. Antibody–antigen complexes were captured by incubation with Protein A/G magnetic beads for 40 min at room temperature. Beads were washed six times with IP wash buffer. Finally, bound proteins were denatured in SDS‐PAGE loading buffer at 95°C for 8 min and stored at −20°C for western blotting analysis.

### 2.14 Immunohistochemistry

Lung tissue sections were fixed in 4% paraformaldehyde for 24 h, dehydrated through a graded ethanol series, and paraffin‐embedded. Sections (4 μm) were dewaxed in xylene and rehydrated in descending ethanol gradients. Antigen retrieval was performed in 1× sodium citrate buffer using a microwave oven followed by 30 min cooling at room temperature. Endogenous peroxidase was blocked with 3% H_2_O_2_ in methanol for 10 min at room temperature. Sections were incubated with primary antibodies at 4°C overnight followed by HRP‐conjugated secondary antibodies. Visualisation was accomplished using 3,3′‐diaminobenzidine (DAB), counterstained with haematoxylin, dehydrated, cleared in xylene, and mounted with neutral balsam. Images were captured using a light microscope and analysed using CaseViewer software.

### 2.15 Immunofluorescence

A549 cells were treated with CB and TGF‐β1 for 24 h. After fixation with 4% paraformaldehyde at room temperature, cells were permeabilised with 0.1% Triton X‐100 in PBS for 5 min. Following three PBS washes, cells were blocked with 5% bovine serum albumin (BSA) at room temperature for 30 min. Cells were incubated with E‐cadherin antibody overnight at 4°C, followed by fluorescent secondary antibodies. After PBST washes, nuclei were counterstained with DAPI. Images were acquired on a Zeiss LSM 800 confocal microscope and quantified with ImageJ.

### 2.16 Molecular Docking

Protein–protein interactions were predicted using AlphaFold3, and predicted structures were analysed with PyMOL v2.3.4 to identify specific amino acid residues at protein–protein interfaces forming hydrogen bonds. These multimer structures were subsequently analysed using PRODIGY to predict binding affinity.

### 2.17 Statistical Analysis

Statistical analyses were performed using GraphPad Prism v8.0. All data were derived from at least three independent experiments and are presented as mean ± SD. Multiple group comparisons were analysed by one‐way ANOVA followed by Tukey's post hoc test; with *p* < 0.05 considered statistically significant.

## 3 Results

### 3.1 Preventive Effect of CB in BLM‐Induced Pulmonary Fibrosis

**FIGURE 1:**
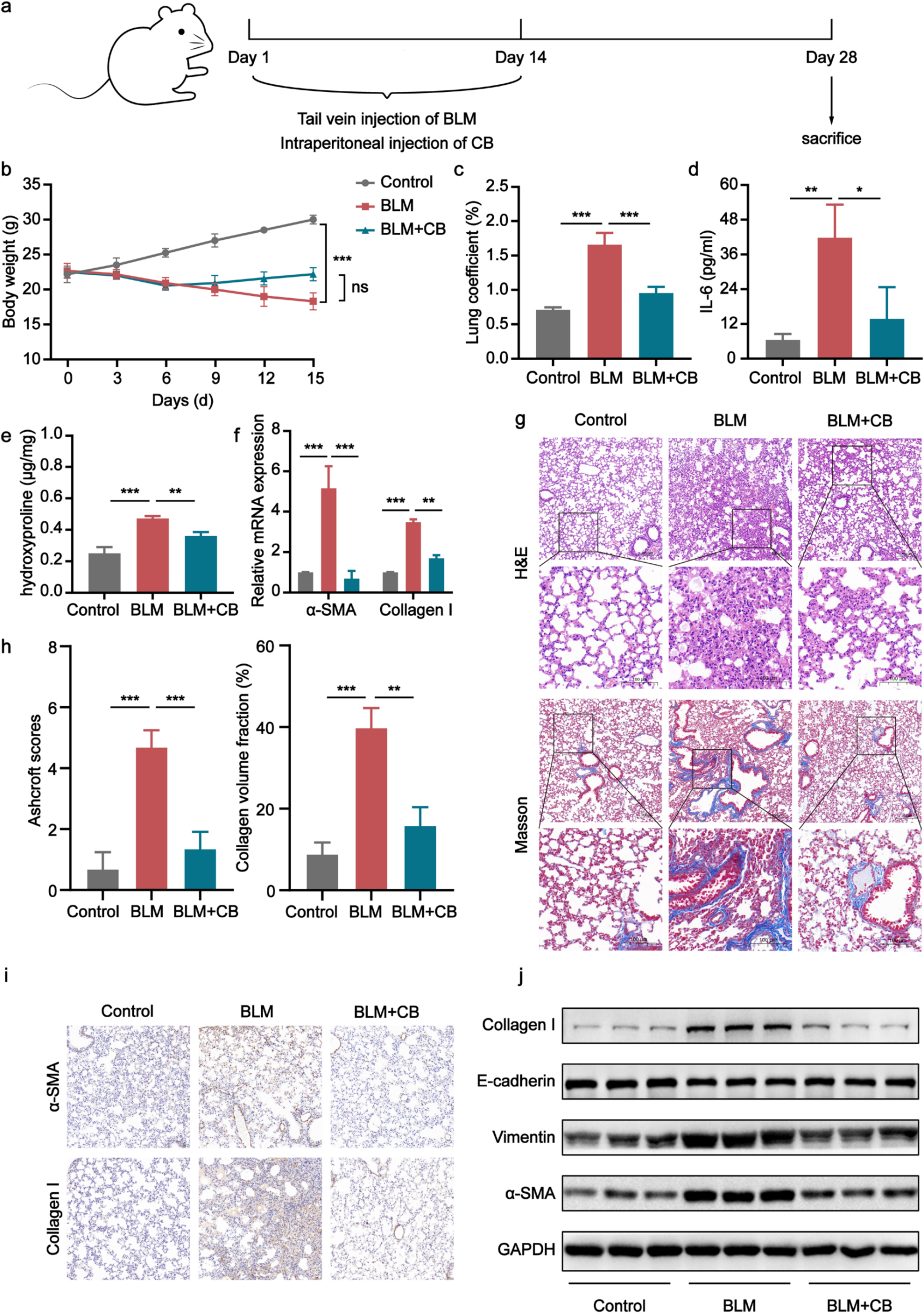
The preventive effect of CB on BLM‐induced pulmonary fibrosis in mice. (a) The sketch of CB in preventing BLM‐induced PF in vivo. (b) Mouse body weight growth curve. (c) Lung coefficient levels of mice in each group. (d) IL‐6 concentrations in mouse serum from each group. (e) Hydroxyproline concentration in mouse lung tissues. (f) qPCR analysis of α‐SMA and collagen I mRNA expression in lung tissues across experimental groups. (g) Representative H&E and Masson's trichrome staining of mouse lung tissues. Scale bars: 100 and 200 μm. (h) Quantitative evaluation of fibrosis severity using Ashcroft scoring and collagen volume fraction. (i) IHC analysis of fibrosis‐related protein expression in tissues. Scale bars: 50 μm. (j) Protein levels of α‐SMA, collagen I, E‐cadherin, and Vimentin in mouse lung tissue homogenates. All data are presented as the mean ± SD (*n* = 3–6). *p < 0.05, **p < 0.01, ***p < 0.001.

To further investigate whether CB influences the progression of PF, a PF model was established by continuous tail vein injection of BLM for 14 days. Starting from the first day of BLM infusion, CB was administered every 3 days, and injections were stopped on day 14. Mice were sacrificed on day 28 to evaluate the preventive effect of CB against BLM‐induced PF in vivo (Figure 1a). Body weight was monitored throughout the experimental period. We discovered that the BLM‐induced model group exhibited reduced body weight gain compared to the control group from days 1 to 14. In contrast, the CB treatment group alleviated the BLM‐induced body weight loss (Figure 1b). Mice in the BLM group showed increased lung coefficients compared to the control group. Conversely, the aforementioned changes were more or less mitigated in the CB treatment group (Figure 1c). Furthermore, serum IL‐6 levels were measured using ELISA. Results demonstrated that mice in the BLM group exhibited significantly elevated serum IL‐6 levels compared to the control group, and the CB treatment group significantly reduced these levels (Figure 1d). HYP, a major component unique to collagen fibres, serves as a biomarker for assessing the severity of PF [28]. HYP content in lung homogenates was significantly elevated in the BLM group compared to the control group. Notably, the CB treatment reduced HYP levels compared to the BLM group (Figure 1e). Myofibroblast activation is a key driver of PF [29], characterised by upregulated expression of fibrotic markers including α‐smooth muscle actin (α‐SMA) and collagen deposition [30]. Western blot and qPCR analyses demonstrated that the CB treatment downregulated collagen I and α‐SMA expression compared to the BLM group. Furthermore, EMT in lung epithelial cells significantly contributes to fibrosis development [31]. The CB treatment also suppressed BLM‐induced EMT markers in vivo, specifically upregulating E‐cadherin and downregulating Vimentin expression (Figure 1f,j). H&E and Masson's staining demonstrated that the BLM group exhibited more inflammatory cell infiltration and unrepaired alveolar cavity destruction and increased collagen deposition compared to the control group. The CB treatment attenuated these BLM‐induced pathological changes, including inflammatory infiltration, alveolar structural damage, and collagen deposition (Figure 1g). Quantitative analysis of PF severity demonstrated elevated Ashcroft scores and collagen volume fraction in the BLM group versus the control group. Crucially, the CB treatment attenuated these alterations (Figure 1h). IHC analysis detected the expression of α‐SMA and collagen I in lung tissues. Compared to the control group, the BLM group exhibited upregulated α‐SMA and collagen I expression. Notably, the CB treatment downregulated α‐SMA and collagen I expression compared to the BLM group (Figure 1i).

### 3.2 Therapeutic Role of CB in BLM‐Induced Pulmonary Fibrosis

**FIGURE 2:**
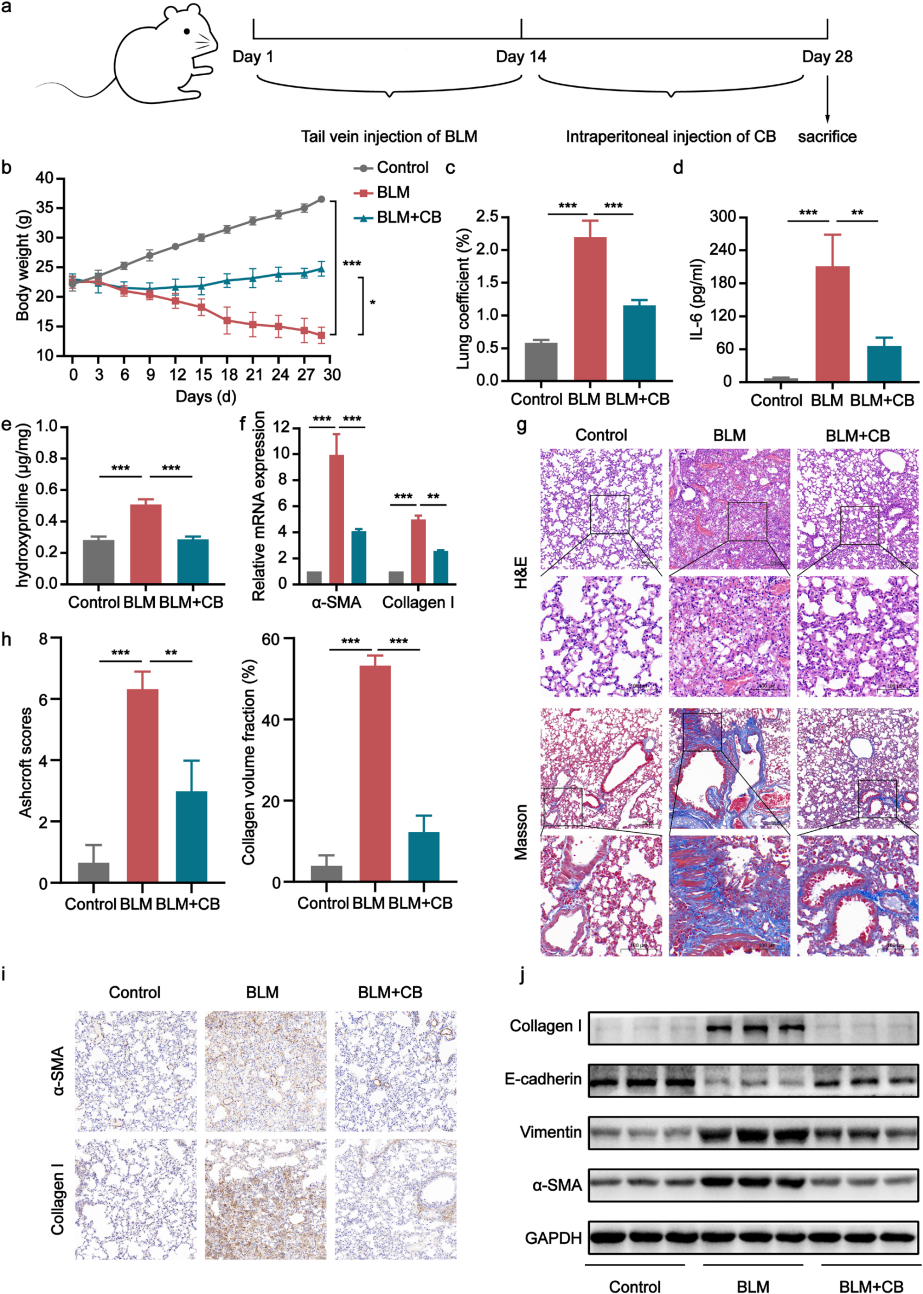
The therapeutic effect of CB on BLM‐induced pulmonary fibrosis in mice. (a) Schematic of the CB‐treated PF model, serum and lung tissue collection at day 28. (b) Mouse body weight growth curve. (c) Graph of lung coefficient levels of mice in each group. (d) The concentration of IL‐6 in the serum of mice in each group. (e) Hydroxyproline levels in lung tissue homogenates of mice in different groups. (f) mRNA levels of α‐SMA and Collagen I were measured in the lung tissues of mice from each experimental group. (g) The H&E and Masson's trichrome staining of the harvested lung samples. Scale bars: 100 and 200 μm. (h) Results of the Ashcroft scores and collagen volume fraction. (i) IHC analysis of α‐SMA and Collagen I expression in lung tissues. Scale bars: 50 μm. (j) Western blot analysis of α‐SMA, Collagen I, E‐cadherin and Vimentin expression. All data are presented as the mean ± SD (*n* = 3–6). *p < 0.05, **p < 0.01, ***p < 0.001.

Based on these findings, a PF model was established via continuous tail vein infusion of BLM for 14 days. Starting on day 14, mice received intraperitoneal injections of CB every 3 days as a therapeutic intervention. This regimen continued until day 28, and serum and lung tissues were collected for analysis (Figure 2a). The BLM‐induced model group showed a significant decrease in body weight when we monitored the body weight of mice from days 1 to 28. In contrast, the CB treatment significantly attenuated this BLM‐induced weight loss compared to the BLM group (Figure 2b). The BLM group exhibited significantly elevated lung coefficients compared to controls, whereas the CB treatment significantly attenuated this BLM‐induced elevation (Figure 2c). These findings indicate that the CB treatment effectively attenuated lung tissue fibrosis and oedema. Serum IL‐6 levels were measured using ELISA. The BLM group exhibited significantly elevated IL‐6 levels compared to controls, whereas the CB treatment significantly reduced IL‐6 levels, indicating that the CB treatment suppressed the inflammatory response (Figure 2d). Quantitative analysis revealed significantly elevated hydroxyproline content in the BLM group compared to controls, while the CB treatment significantly reduced hydroxyproline levels compared to the BLM group, approaching control levels (Figure 2e). Western blot analysis demonstrated that the CB treatment significantly upregulated E‐cadherin expression while downregulating α‐SMA, Collagen I, and Vimentin protein levels. These changes were corroborated by qPCR analysis of corresponding mRNA expression (Figure 2f,j). Consistently, H&E and Masson's staining of lung sections demonstrated that the CB treatment attenuated BLM‐induced histopathological changes, including structural fibrosis and collagen deposition (Figure 2g). Similarly, Ashcroft scores and collagen volume fraction demonstrated a significant reduction in fibrosis severity following the CB treatment compared to the BLM group (Figure 2h). Finally, IHC analysis demonstrated that the CB treatment significantly attenuated BLM‐induced upregulation of key lung fibrosis markers in lung tissues (Figure 2i).

### 3.3 CB Suppresses TGF‐β1‐Induced Fibroblast Growth and Activation In Vitro

**FIGURE 3:**
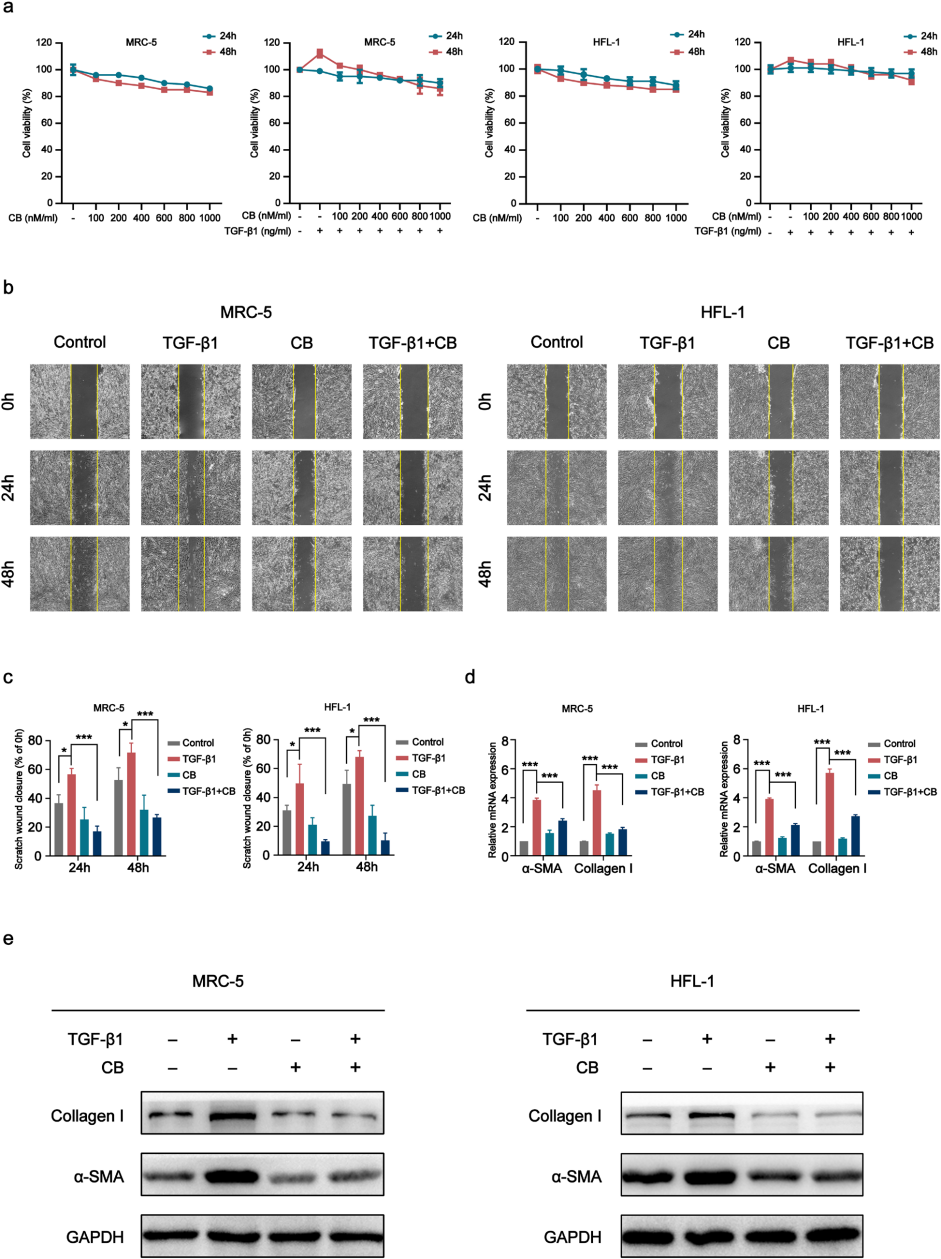
CB inhibits TGF‐β1‐induced fibroblast proliferation and activation in vitro. (a) Cell viability plots of MRC‐5 and HFL‐1 cells in the presence or absence of TGF‐β1. (b) Representative images of scratched MRC‐5, HFL‐1 cells treated with TGF‐β1 and CB. Scale bar: 100 μm. (c) Quantitative analysis of MRC‐5 and HFL‐1 cell scratches. (d) The mRNA levels of α‐SMA and collagen I in cells according to qPCR analysis. (e) Western blot was conducted to quantify α‐SMA and collagen I expression in cells treated with TGF‐β1 and CB for 48 h. All data are presented as the mean ± SD. *p < 0.05, ***p < 0.001.

When fibroblasts' homeostatic potential is interrupted, they activate and convert into myofibroblasts [32]. First, cells were treated with CB at various concentrations for 24 or 48 h, in the presence or absence of TGF‐β1, to determine whether CB is cytotoxic. The results demonstrated that CB treatment did not induce cytotoxicity at the tested concentrations (Figure 3a). TGF‐β1 significantly induced the migration of both MRC‐5 and HFL‐1 cells in scratch assays. CB treatment attenuated this TGF‐β1‐induced migration. Furthermore, quantitative analysis revealed delayed wound healing in CB‐treated groups compared to TGF‐β1‐induced groups, indicating that CB suppressed the migration of cells (Figure 3b,c). Western blot and qPCR analyses demonstrated that CB reduced the expression levels of myofibroblast markers Collagen I and α‐SMA, indicating that CB inhibits fibroblast‐to‐myofibroblast differentiation (Figure 3d,e).

### 3.4 CB Inhibits TGF‐β1‐Induced EMT in A549 Cells

**FIGURE 4:**
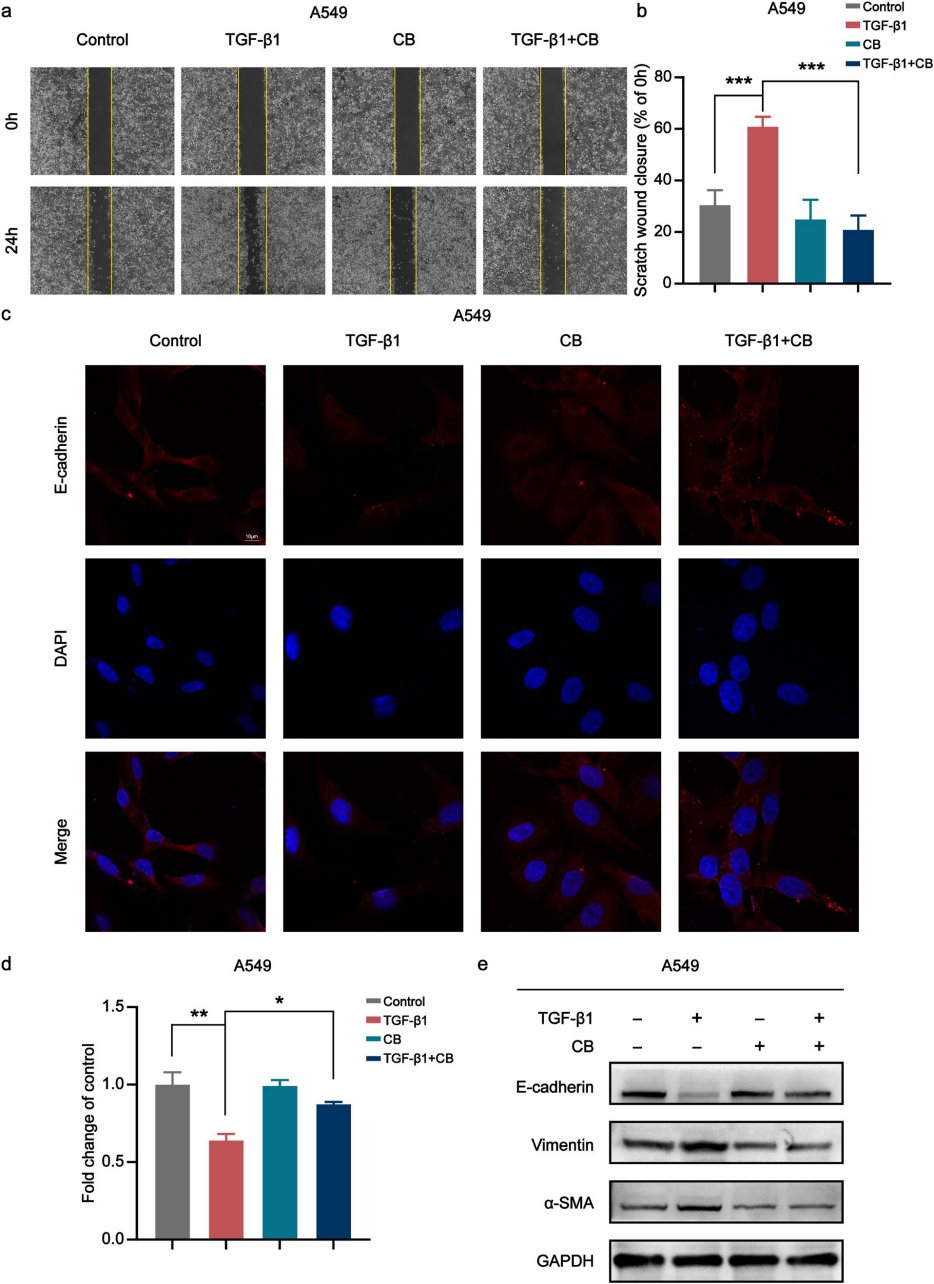
CB suppresses TGF‐β1‐induced EMT process in A549 cells. (a) Representative images of the effects of TGF‐β1 and CB treatments on scratch healing in A549 cells. Scale bar: 100 μm. (b) Quantitative analysis of scratch healing in A549 cells. (c) Representative image of E‐cadherin immunofluorescence in A549 cells. Scale bar: 10 μm. (d) Quantitative results of E‐cadherin immunofluorescence intensity. (e) Western blot analysis of E‐cadherin, α‐SMA, and Vimentin protein expression. All data are presented as the mean ± SD. *p < 0.05, **p < 0.01, ***p < 0.001.

TGF‐β1 induces tissue fibrogenesis and EMT—a key mechanism in PF pathogenesis [33]. This experimental model for EMT induction in A549 epithelial cells is well‐established. Therefore, we further evaluated the effect of CB on EMT in A549 cells. We observed that TGF‐β1 significantly enhanced A549 cell migration into the wound area, while CB attenuated this TGF‐β1‐induced migration in A549 cells (Figure 4a,b). Immunofluorescence results showed that CB treatment enhanced the expression of E‐cadherin in A549 cells (Figure 4c). In addition, quantitative analysis confirmed significantly elevated E‐cadherin levels in CB‐treated versus TGF‐β1‐treated cells (Figure 4d). Western blot analysis revealed that TGF‐β1 treatment significantly decreased the expression of E‐cadherin in A549 cells, while upregulating the protein levels of α‐SMA and Vimentin. CB treatment significantly reversed these TGF‐β1‐induced changes (Figure 4e).

### 3.5 RNA‐Seq Analysis of CB Treatment in a BLM‐Induced Lung Fibrosis Model

**FIGURE 5:**
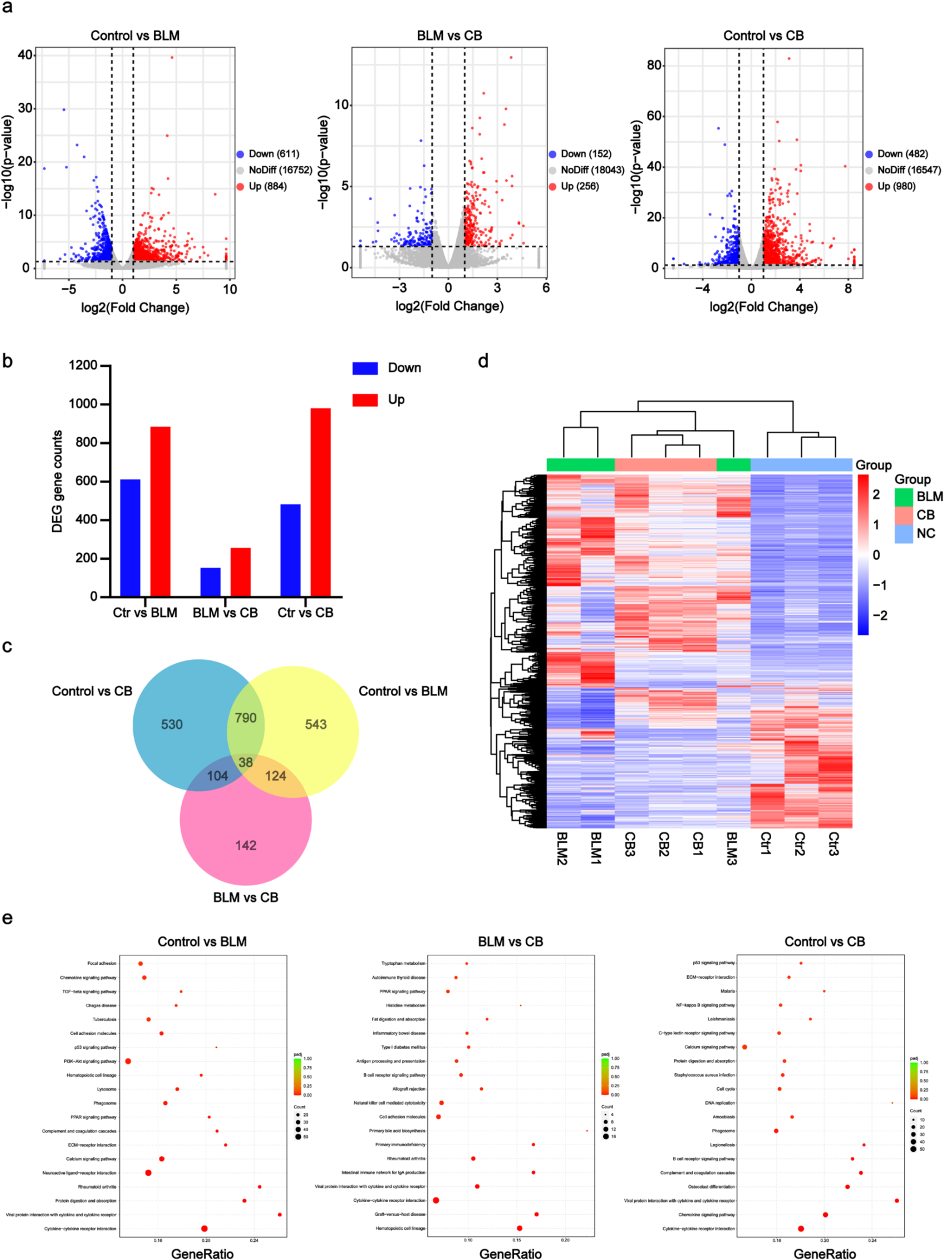
RNA‐seq analysis of CB treatment in BLM‐induced PF model. (a) Volcano plots of DEGs: Control versus BLM, BLM versus CB, Control versus CB. (b) Bar graph of upregulated and downregulated DEGs counts. (c) Venn diagram of overlapping DEGs among experimental groups. (d) Hierarchical clustering heatmap of DEGs across experimental groups. (e) KEGG pathway analysis of primary signalling pathways.

In this study, we investigated the effect of CB treatment on gene expression in the BLM‐induced mouse lung fibrosis model using RNA‐seq, and identified differentially expressed genes among the three groups, which are visualised in the volcano plot (Figure 5a). The numbers of upregulated and downregulated DEGs are visualised in the bar graph (Figure 5b). The Venn diagram illustrates the number of overlapping DEGs between the Control vs. BLM, BLM vs. CB, and Control versus CB comparison groups (Figure 5c). The number of shared DEGs among the three comparison groups was quantified. Additionally, the heatmap displays the hierarchical clustering pattern of DEGs across all experimental groups and their intergroup expression differences (Figure 5d). KEGG pathway analysis identified the primary signalling pathways: the PPAR signalling pathway, NF‐κB signalling pathway, TGF‐β signalling pathway, PI3K‐Akt signalling pathway and p53 signalling pathway (Figure 5e). The top 20 most significantly enriched pathways were selected for visualisation in the graph.

### 3.6 Silencing PI15 Suppresses TGF‐β1‐Induced Fibroblast Activation and Related Gene Expression

**FIGURE 6:**
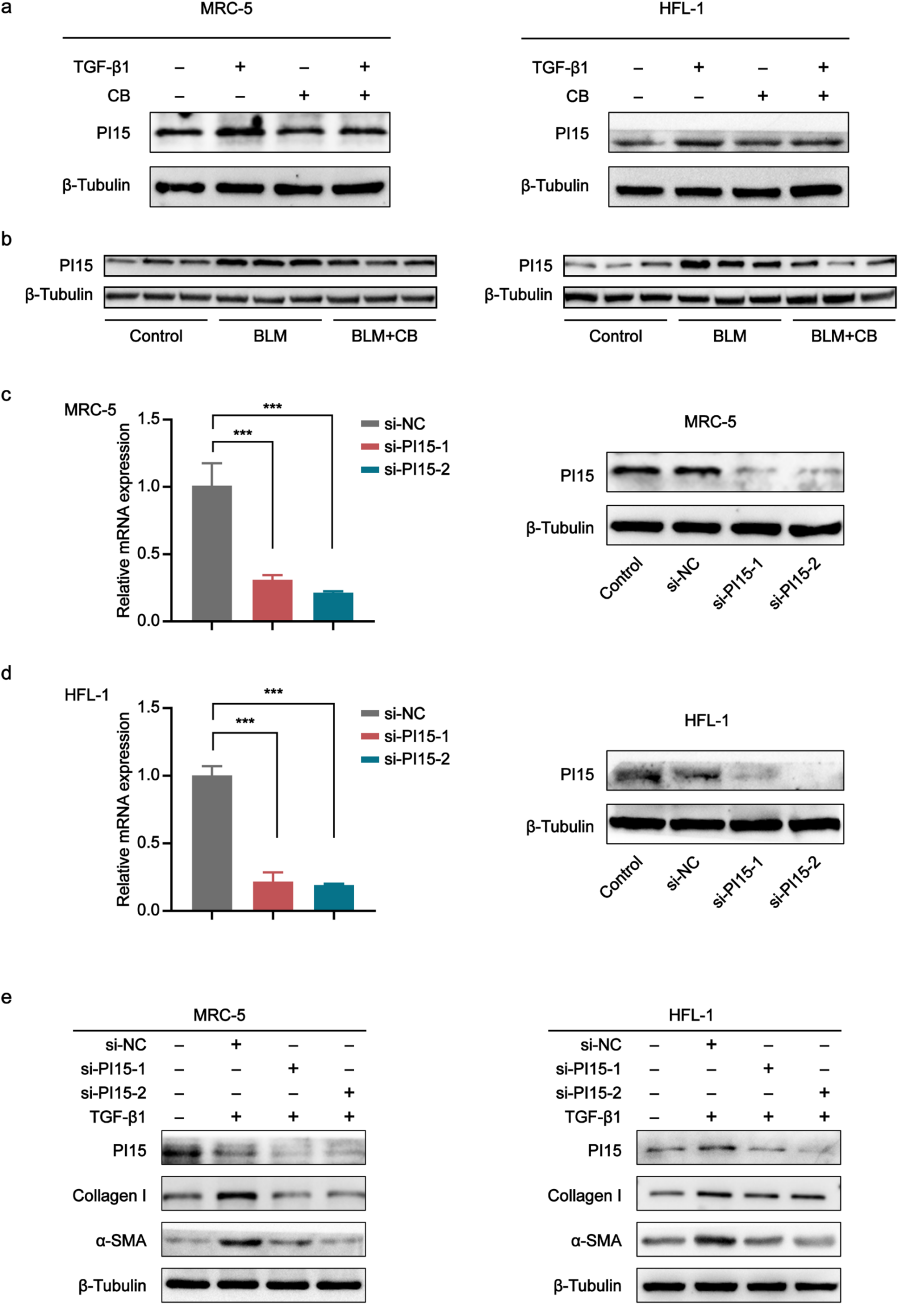
Silencing PI15 suppresses TGF‐β1‐induced PF progression. (a) Western blot analysis of PI15 expression in TGF‐β1‐induced MRC‐5 and HFL‐1 cells following CB treatment. (b) Western blot analysis of PI15 expression in lung tissues from BLM‐induced mice administered CB. (c) Validation of siRNA transfection efficiency in MRC‐5 cells by western blot and qPCR. (d) Validation of siRNA transfection efficiency in HFL‐1 cells by western blot and qPCR. (e) Western blot analysis of α‐SMA and Collagen I protein expression following silencing PI15. All data are presented as the mean ± SD (*n* ≥ 3). *p < 0.05, **p < 0.01, ***p < 0.001.

Through high‐throughput RNA‐seq analysis of mouse lung tissues, PI15 expression was significantly upregulated in PF, prompting further investigation of this gene. In MRC‐5 and HFL‐1 cells treated with identical concentrations of TGF‐β1 and CB for 48 h using the same methods, PI15 protein levels were quantified. Notably, TGF‐β1 stimulation significantly upregulated PI15 protein levels, whereas CB treatment significantly suppressed PI15 protein expression (Figure 6a). We further validated in mouse lung tissues that PI15 protein levels were significantly elevated in both BLM‐induced prevention and therapeutic treatment groups, whereas CB treatment significantly suppressed PI15 protein expression (Figure 6b). These findings collectively demonstrate that PI15 is upregulated in PF. Consequently, we investigated the impact of PI15 silencing on TGF‐β1‐induced PF. Subsequently, the silencing efficiency of PI15 siRNA was assessed by western blot and qPCR. The results confirmed that siPI15‐1 and siPI15‐2 were two efficient knockdowns targeting PI15 (Figure 6c,d). Concurrently, the expression of myofibroblast markers α‐SMA and Collagen I was significantly downregulated after PI15 silencing using siRNA (Figure 6e).

### 3.7 The Differential Gene PI15 Interacts With TGF‐β1 and Suppresses the TGF‐β/Smad Signalling Pathway

**FIGURE 7:**
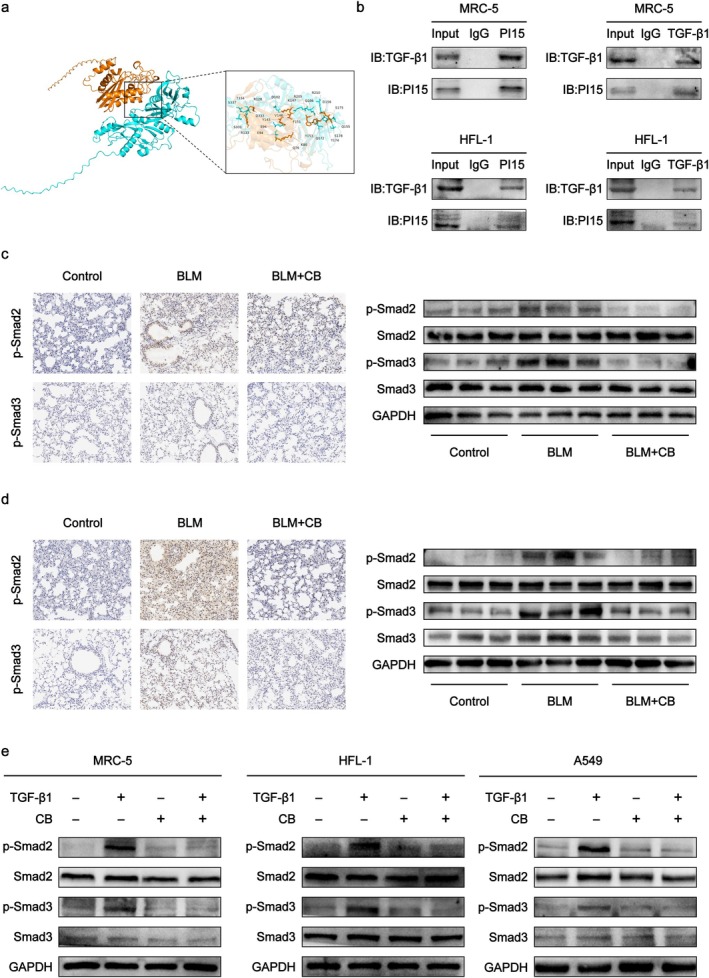
The effect of the differential gene PI15 on the TGF‐β1/Smad signalling pathway. (a) Molecular docking results for PI15 and TGF‐β1. (b) Co‐IP assay demonstrated the interaction between PI15 and TGF‐β1. (c) Results of p‐Smad3 and p‐Smad2 detection in lung tissue sections and lung homogenates of mice in the prevention group. Scale bar: 100 μm. (d) Results of p‐Smad3 and p‐Smad2 detection in lung tissue sections and lung homogenates of mice in the treatment groups. Scale bar: 100 μm. (e) Western blot was conducted to assess the protein expression of Smad3, p‐Smad3, Smad2 and p‐Smad2 in MRC‐5, HFL‐1, and A549 cells.

Molecular docking revealed a PI15‐TGF‐β1 binding energy of −11.2 kcal/mol, indicating a high‐affinity interaction between the two proteins (Figure 7a). Subsequently, Co‐IP experiments were conducted to verify the interaction between PI15 and TGF‐β1 in MRC‐5 and HFL‐1 cells. Results demonstrated their interaction (Figure 7b). TGF‐β1 plays a vital role in tissue fibrosis by stimulating the downstream Smad signalling pathway [34, 35, 36]. As detected by IHC and western blot, BLM significantly increased p‐Smad2 and p‐Smad3 levels in mouse lung tissues, whereas CB suppressed their phosphorylation (Figure 7c,d). Western blot analysis of p‐Smad2 and p‐Smad3 expression, as shown in (Figure 7e), revealed significant upregulation of p‐Smad2 and p‐Smad3 in TGF‐β1‐stimulated MRC‐5, HFL‐1 and A549 cells, whereas CB intervention effectively suppressed phosphorylation of these proteins.

## 4 Discussion

PF is a chronic, progressive lung disorder in which alveolar structures are progressively replaced by fibrous tissue, ultimately leading to respiratory failure. This condition poses a serious threat to human health. Currently, approved pharmacotherapies for PF can only delay disease progression but cannot halt the fibrotic process [37, 38]. Although several candidate drugs are under investigation, their therapeutic efficacy remains unconfirmed. Accumulating evidence indicates shared pathophysiological mechanisms between PF and lung cancer, encompassing EMT, dysregulated cell proliferation/differentiation, aberrant intercellular communication, inflammatory responses, tissue invasion, and signalling pathway abnormalities [39, 40, 41]. In recent years, the strategy of using old drugs in new ways has gained increasing attention. Although CB exhibits diverse antitumor activities, its anti‐fibrotic mechanisms remain underexplored. Therefore, we selected CB to elucidate its therapeutic efficacy against PF and the underlying molecular mechanisms.

Currently, the BLM‐induced murine model is one of the most critical animal models in PF research [42], characterised by histopathological features including inflammatory cell infiltration, collagen deposition, fibrotic nodule formation, alveolar wall thickening, alveolar architectural destruction and fibroblast proliferation [43]. To evaluate CB's preventive effects on PF, we established a murine PF model through daily tail vein injections of BLM (10 mg/kg) for 14 days with concomitant intraperitoneal administration of CB (4 mg/kg) every third day from days 1 to 14; to assess therapeutic efficacy, an identical BLM‐induced model was established, followed by CB administration via intraperitoneal injection every 3 days from day 14 to day 28. This study demonstrates that CB confers significant preventive and therapeutic efficacy in the BLM‐induced murine PF model. CB administration significantly restored lung HYP content, suppressed aberrant collagen deposition, attenuated inflammatory responses and ameliorated alveolar structural damage including septal thickening. These findings reveal the potential of CB as a therapeutic candidate for PF for the first time.

TGF‐β1 acts as a ‘master switch’ in PF progression, driving fibrogenesis through initiation of fibrosis, alveolar epithelial cell injury, myofibroblast activation, and EMT [44, 45]. Crucially, sustained TGF‐β1‐mediated myofibroblast activation potently accelerates fibrotic progression [46]. While EMT's role in PF remains debated, substantial evidence confirms TGF‐β's capacity to induce EMT in pulmonary parenchymal cells [47]. This study demonstrated that CB significantly suppressed α‐SMA and Collagen I expression in TGF‐β1‐stimulated fibroblasts, while inhibiting TGF‐β1‐induced cell migration ability. In summary, CB ameliorated BLM‐induced PF in vivo and suppressed TGF‐β1‐stimulated fibroblast activation and EMT in vitro. We next delineated the molecular mechanisms underlying the above effects of CB.

High‐throughput transcriptome sequencing of murine lung tissues from three treatment groups identified 38 overlapping DEGs across all groups. Key upregulated DEGs comprised Spp1, Retnla, Saa3, Orm1, Clca3a1, Dio3, PI15, Timp1, Ereg, Col28a1, Prss22, Sorcs1 and Gal3st2; notable downregulated DEGs included Cidec, Colq, Krt79, Fabp1, Faim2, Cd300e, Slc7a10, Slc6a4, Ccr3, Wfdc16 and Pck1. We are particularly interested in PI15, identified as significantly DEGs in our RNA‐Seq analysis. Existing evidence indicates PI15 is highly expressed in a variety of tumour tissues and is a trypsin‐binding protein [48]. Nevertheless, its role in PF pathogenesis remains poorly defined. Following PI15 knockdown, we observed that genetic silencing of PI15 significantly attenuated TGF‐β1‐induced fibroblast activation, as demonstrated by reduced protein expression of α‐SMA and Collagen I. These results demonstrate that PI15 knockdown effectively suppresses myofibroblast differentiation and pathological collagen deposition.

Furthermore, we employed the BioGRID (https://thebiogrid.org/) database to identify potential protein interactors of PI15. Subsequently, molecular docking simulation analysis of PI15 and TGF‐β1 showed that the binding energy between the two was −11.2 kcal/mol, indicating that they have good binding potential. Co‐IP assays demonstrated binding between PI15 and TGF‐β1 in both MRC‐5 and HFL‐1 fibroblast lines. Prior studies establish TGF‐β1 as a master regulator of tissue fibrosis, driving fibrogenesis primarily through canonical Smad signalling pathway activation [49]. Specifically, Smad2 hyperphosphorylation and Smad3 hyperphosphorylation play pivotal roles in promoting fibrotic responses [50]. We therefore investigated whether CB's anti‐fibrotic effects involve TGF‐β/Smad pathway inhibition. Our data demonstrate that CB effectively suppresses TGF‐β1‐induced Smad2 and Smad3 phosphorylation, thereby inhibiting PF progression.

## 5 Conclusion

In summary, this study demonstrates that CB significantly ameliorates BLM‐induced PF in vivo while suppressing TGF‐β1‐stimulated fibroblast activation and EMT in vitro. Furthermore, PI15 serves as a critical target through which CB exerts its anti‐PF effects, and its mechanism likely involves inhibiting the activation of the TGF‐β/Smad signalling pathway. This study provides novel insights into the theoretical foundation for PF prevention and treatment.

## Author Contributions

**Dong Xia:** formal analysis (equal), investigation (equal), methodology (equal), validation (equal), visualization (equal), writing – original draft (equal). **Xingyan Liu:** investigation (equal), methodology (equal), validation (equal), visualization (equal), writing – review and editing (equal). **Li Li:** data curation (equal), investigation (equal), methodology (equal). **Yong You:** methodology (equal), validation (equal). **Jing Wang:** funding acquisition (equal), investigation (equal), project administration (equal), supervision (equal), writing – review and editing (equal). **Weiyi Fang:** funding acquisition (equal), resources (equal), supervision (equal), writing – review and editing (equal). **Huiling Yang:** conceptualization (equal), data curation (equal), funding acquisition (equal), project administration (equal), supervision (equal), writing – review and editing (equal).

## Ethics Statement

This study was reviewed and approved by the Experimental Animal Ethics Committee of Guangdong Medical University (GDY2302460).

## Conflicts of Interest

The authors declare no conflicts of interest.

## Data Availability

The data that support the findings of this study are available on request from the corresponding author. The data are not publicly available due to privacy or ethical restrictions.
